# A Lot of Action, But Not in the Right Direction: Systematic Review and Content Analysis of Smartphone Applications for the Prevention, Detection, and Management of Cancer

**DOI:** 10.2196/jmir.2661

**Published:** 2013-12-23

**Authors:** Jacqueline Lorene Bender, Rossini Ying Kwan Yue, Matthew Jason To, Laetitia Deacken, Alejandro R Jadad

**Affiliations:** ^1^ELLICSR Health, Wellness and Cancer Survivorship CentrePrincess Margaret Cancer CentreUniversity Health NetworkToronto, ONCanada; ^2^Centre for Global eHealth InnovationToronto General HospitalUniversity Health NetworkToronto, ONCanada; ^3^Dalla Lana School of Public HealthUniversity of TorontoToronto, ONCanada; ^4^Universite MontpellierMontpellierFrance

**Keywords:** mobile, Internet, cancer, software applications, apps

## Abstract

**Background:**

Mobile phones have become nearly ubiquitous, offering a promising means to deliver health interventions. However, little is known about smartphone applications (apps) for cancer.

**Objective:**

The purpose of this study was to characterize the purpose and content of cancer-focused smartphone apps available for use by the general public and the evidence on their utility or effectiveness.

**Methods:**

We conducted a systematic review of the official application stores for the four major smartphone platforms: iPhone, Android, Nokia, and BlackBerry. Apps were included in the review if they were focused on cancer and available for use by the general public. This was complemented by a systematic review of literature from MEDLINE, Embase, and the Cochrane Library to identify evaluations of cancer-related smartphone apps.

**Results:**

A total of 295 apps from the smartphone app stores met the inclusion criteria. The majority of apps targeted breast cancer (46.8%, 138/295) or cancer in general (28.5%, 84/295). The reported app purpose was predominantly to raise awareness about cancer (32.2%, 95/295) or to provide educational information about cancer (26.4%, 78/295), followed by apps to support fundraising efforts (12.9%, 38/295), assist in early detection (11.5%, 34/295), promote a charitable organization (10.2%, 30/295), support disease management (3.7%, 11/295), cancer prevention (2.0%, 6/295), or social support (1.0%, 3/295). The majority of the apps did not describe their organizational affiliation (64.1%, 189/295). Apps affiliated with non-profit organizations were more likely to be free of cost (χ^2^_1_=16.3, *P*<.001) and have a fundraising or awareness purpose (χ^2^_2_=13.3, *P*=.001). The review of the health literature yielded 594 articles, none of which reported an evaluation of a cancer-focused smartphone application.

**Conclusions:**

There are hundreds of cancer-focused apps with the potential to enhance efforts to promote behavior change, to monitor a host of symptoms and physiological indicators of disease, and to provide real-time supportive interventions, conveniently and at low cost. However, there is a lack of evidence on their utility, effectiveness, and safety. Future efforts should focus on improving and consolidating the evidence base into a whitelist for public consumption.

## Introduction

Since the beginning of the 21st century, mobile phones have become nearly ubiquitous. At the end of 2011, there were an estimated 6 billion mobile subscriptions, accounting for approximately 87% of the global population [1]. Rapid technological convergence has led to the emergence of smartphones—feature-rich phones that combine the voice and text messaging functions of basic phones with powerful computing technology that can support third-party applications, sensing, Internet access, and wireless connectivity with other devices. According to a 2012 report from the Pew Internet and American Life Project, 85% of US adults own a cell phone of some kind and 53% own a smartphone [2]. The combination of their popularity, technical capabilities, and proximity to their owners makes them an attractive platform for the delivery of health promotion and disease management interventions [3].

Basic mobile phone-based interventions have shown promise in improving outcomes for a variety of health conditions and behaviors. A systematic review of controlled trials of health care interventions delivered by cell phones with basic features such as voice or text-messaging capabilities reported improvements in 61% of outcomes measured [4]. Process improvements included improved attendance at medical appointments, quicker time to diagnosis and treatment, and enhanced communication skills. Behavioral changes included smoking cessation, improved medication adherence, and more timely vaccinations. Clinically significant changes included improvements in blood sugar control, asthma symptoms, stress levels, and self-efficacy. Most of these mobile interventions used “push” technology, where participants received personalized text or automated voicemail messages such as appointment, medication, or symptom assessment reminders, or educational messages to encourage preventive health behaviors or self-management activities.

Less is known about smartphone-based interventions, which offer new possibilities for health promotion and disease management. Technical capabilities include: text messaging, cameras, Internet access, automated sensing, and native applications. All major smartphone platforms—Apple iOS, Google Android, BlackBerry, Nokia’s Symbian, and Nokia and Microsoft Windows Phone—provide third-party developers with application programming interfaces (APIs) that they can use to build special purpose applications, known as native applications (apps). The launch of the Apple App Store in 2008, along with its software development kit shortly thereafter, led to an explosion in app development and subsequent downloads. As of April 2012, the total number of consumer health apps for the iPhone was estimated to be 13,600 [5]. Klasjna and Pratt [2] have identified five strategies used in smartphone-based health interventions that take advantage of the technical capabilities of the mobile phone to various extents; these include: (1) tracking health information (eg, through text messaging, native apps, or automated sensing), (2) involving the health care team (eg, remote coaching, symptom monitoring), (3) leveraging social influence (eg, peer-to-peer support, modeling, or influence), (4) increasing the accessibility of health information (eg, short messages or reminders), and (5) utilizing entertainment (eg, games to motivate health management).

Researchers have begun to characterize the smartphone apps that are available to consumers for pain management [6], diabetes management [7], smoking cessation [8], cancer [9], and melanoma detection [10]. Cancer is a leading cause of death and disability worldwide, accounting for 7.6 million deaths (13% of all deaths) in 2008 [11]. Recent advances in detection, prevention, and treatment have led to increased survival rates for patients living with the disease, with an estimated 28 million survivors around the world [12]. A large majority of cancer survivors experience distressing symptoms and adverse long-term consequences related to their disease and unmet supportive care needs are frequently reported [13]. As a result, many people affected by cancer regularly seek health-related information online [14-16]. The review of cancer apps by Pandey et al [9], although informative, was restricted to those available on the iPhone market and lacked detail on the content or features of apps. For example, consumer apps were described as “general information about the disease” or “patient assistant tools”.

The purpose of this study was to characterize the purpose and content of cancer-focused smartphone apps available for use by the general public and the evidence on their utility or effectiveness. We present a systematic review and characterization of the cancer-focused apps that are available in four leading smartphone application stores. This was complemented by a systematic review of the health literature to identify evaluations of cancer-focused smartphone apps. We also discuss the reported purpose and features of smartphone apps in relation to health behavior change theory, with the goal of identifying gaps, which could inform intervention development.

## Methods

### Overview

Systematic review methodology, as described by Moher et al [17], was used to guide the collection and characterization of eligible apps from the official smartphone stores and the evidence on app utility or effectiveness from the health literature. We developed a systematic search strategy that attempted to identify all relevant apps and studies and we provide a systematic presentation and synthesis of the characteristics of the apps and the studies.

### Mobile Application Market Search

#### Overview

On February 14, 2012, we conducted a search of the online stores for iPhone (App Store), Android (Google Play), BlackBerry (App World), and Nokia**/**Symbian (Ovi) using the keyword “cancer” in the main search engine. We searched across all store categories (eg, we did not restrict our search to the health/ lifestyle category). We restricted our search to applications that had “cancer” in the title or store description of the app. One author (LD) searched the Android and BlackBerry markets in France and another author (RY) searched the iPhone and Nokia markets for eligible apps in Canada. All eligible apps from the French Android and BlackBerry stores were checked against the Canadian Android and BlackBerry stores to ensure their availability in the Canadian market. The two reviewers then swapped a random selection of 5% (a total of 64 apps) of their search yields to verify their eligibility.

#### Selection Criteria

Apps were included if they were focused on cancer and were intended for people affected by cancer defined as cancer patients or survivors, their family caregivers, or the general public concerned about cancer; had an English-language interface; and were available for smartphones. If an app had an English interface, the app, and its title and description appeared in English regardless of the store in which the search was conducted. Some apps were available in multiple languages. Apps were excluded if they were only available on tablet computers or were aimed at health care professionals. We excluded apps related to smoking cessation, radiation exposure, or general symptom management because they were not focused on cancer and many did not include “cancer” in their title or store description. Moreover, recent reviews have been published on smoking cessation apps [8] and pain management apps [6]. Inter-rater reliability of the 64 apps (5%) reviewed for eligibility as determined by standard Cohen’s kappa was acceptable (.74). Disagreements were resolved by consensus involving a third reviewer when necessary.

#### Data Extraction

We collected information from the store description of the app and only downloaded or examined the websites of those apps that had unclear store descriptions or did not provide screenshots. We extracted information on: year of release, cost, affiliation (eg, commercial, non-profit, university, or medical center), condition information (eg, type of cancer), source of app information, features (eg, calendar, journaling software, etc), and multimedia used (eg, text, audio, visual, video).

#### Data Coding and Analysis

We generated a preliminary coding scheme to describe the purpose of the app by collectively (JLB, RY, LD) analyzing the content of the first 50 (16.2%, 50/309) eligible apps. Using this coding scheme, one author (RY) extracted the study data and coded the main purpose of the apps. Another author (MT) independently reviewed a random selection of 25.6% (79/309) of apps to verify the information coded. Inter-rater reliability of the coded data, as determined by Cohen’s kappa was high (.87), but revealed that minor changes to the coding scheme were required. RY used the revised coding scheme to re-code all apps. The final coding scheme was based on the following seven identified categories of apps:

1. Awareness-raising: tools to raise public recognition of cancer as a societal problem.
2. Fundraising: tools to attract financial resources for cancer control.
3. Promote an organization: encourage awareness about a charitable organization raising awareness and funds for cancer or providing support to people affected by cancer.
4. Disease and treatment information: provide general information about cancer (eg, disease or treatment options)
5. Prevention: provide information and practical tools to avoid cancer, including the recurrence of cancer.
6. Early detection: provide information and tools to assist in the identification of cancer before the emergence of symptoms or signs.
7. Disease management: provide information and practical tools to deal with the medical, behavioral, or emotional aspects of cancer.
8. Support: provide access to peer or professional assistance.

Apps were coded into one category based on their main purpose as described in the store description.

### Health Literature Search

#### Overview

Articles were identified through a search of MEDLINE (1990-June 18, 2012), Embase (1990-June 24, 2012), and all of the databases in the latest available version of The Cochrane Library (1990-June 24, 2012). The search strategy, developed in consultation with a medical librarian (ME), included a string of mobile technology search terms cross-matched with terms “cancer” and “neoplasm” (Multimedia Appendix 1). The yield from the bibliographic databases was supplemented with a review of reference lists from eligible articles and recent reviews. Two of the authors of this study (MT and JLB) independently reviewed the titles and abstracts of the total search yield to identify eligible articles. The full text of the article was retrieved if any reviewer considered a citation potentially relevant.

#### Selection Criteria

Articles were considered potentially relevant if they: described an evaluation of a mobile phone app for cancer patients/survivors, family caregivers, or the general public; included original data on the use of the mobile phone app by cancer patients/survivors, family caregivers, or the general public; and were published in English. To be eligible for inclusion in the final analysis, the article must have described an evaluation of a cancer-focused smartphone app. We excluded articles that described or evaluated basic mobile phone and personal digital assistant (PDA) interventions, the reliability of paper versus mobile phone-based assessments, as well as articles that evaluated apps tested exclusively on laptops, netbooks, or tablet computers. As described, a smartphone is a feature-rich phone that offers more computing capability than a basic mobile phone or a PDA.

#### Data Extraction and Coding

Two of the authors of this study (MT and JLB) independently reviewed the full text of the articles meeting the eligibility criteria and extracted information on the following: general study characteristics (eg, primary author, year of publication, country of study, and source of funding); participants (age, gender, and sociodemographic data); condition (eg, cancer type); intervention (eg, device, main purpose, and features); study design, and findings. Disagreements were rare and were easily resolved by consensus.

## Results

### General Characteristics

**Figure 1:**
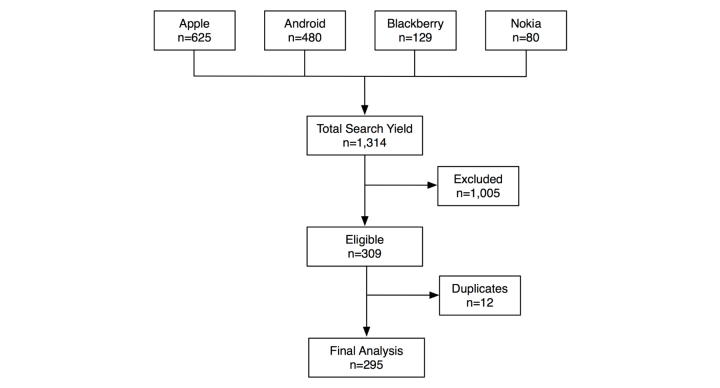
Flow diagram.

**Figure 2:**
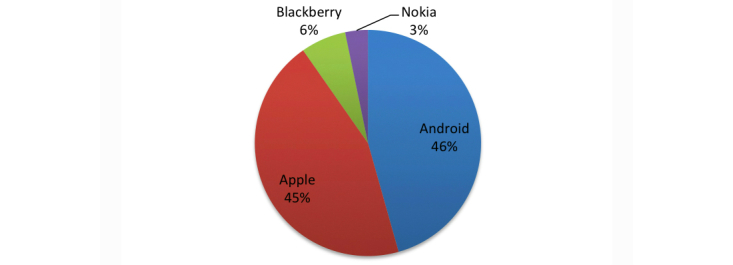
Distribution of cancer apps across the four mobile markets.

The search of the mobile phone market yielded 1314 potentially relevant apps, of which 309 apps met our selection criteria (Figure 1); 90.3% (279/309) of apps were available on the iPhone or Android markets (Figure 2). Twelve apps were available on more than one platform (10 were available in two stores and 2 in three stores). Therefore, there were a total of 295 unique apps.

Release date information was available for only 38.0% (112/295) of the apps from Apple, Android, and BlackBerry, as the remainder had produced updated versions and only published their date of update. Release date information was not available for apps on the Nokia market.

Half of the apps (50.2%, 148/295) were free to download. Of those free-to-download apps, 8 were trial versions of the full pay-for-download applications. These free apps offered limited versions of the full apps, restricting access to the full suite of features. The remainder of the apps (47.1%, 139/295) ranged from $1 to $12 CAD and the majority were priced at a median of $1.01 CAD.

The majority of the apps did not describe their organizational affiliation (64.1%, 189/295). Of those that provided organizational information, 63.2% (67/106) were affiliated with a non-profit, 26.4% (28/106) with a commercial company (eg, Health Monitor Network), 9.4% (10/106) with a university or medical institution, and 1 app was affiliated with a government institution (eg, National Institutes for Health). Apps affiliated with not-for-profit organizations (non-profit, university, medical institution, or government) were more likely to be free (χ^2^_1_=16.3, *P*<.001). Apps that did not disclose their affiliation were more likely to have a price (χ^2^_1_=50.1, *P*<.001).

### Target Cancer Type

**Figure 3:**
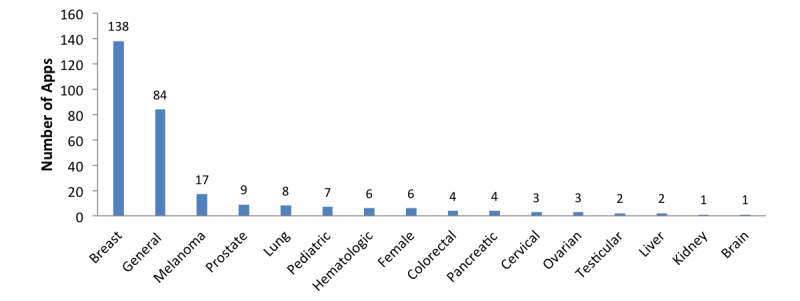
Number of apps by target cancer type.

**Figure 4:**
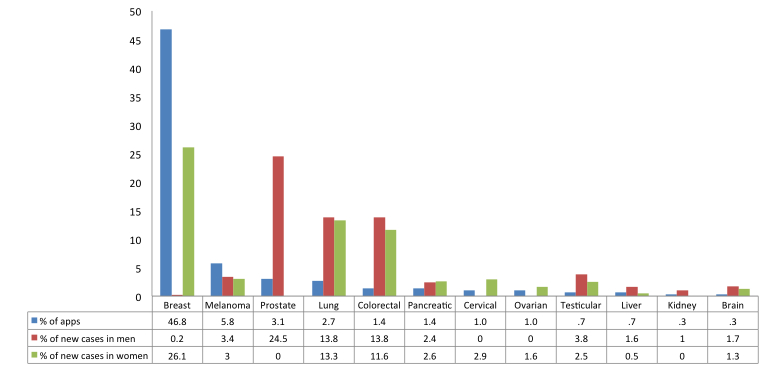
Percent distribution of apps in comparison to estimated new cases of cancer, by gender, Canada 2013. Data on cancer incidence drawn from Figure 1.2 of Canadian Cancer Statistics by the Canadian Cancer Society (2013).

Overall, the apps targeted 15 types of cancer, as well as broadly targeting pediatric cancers, female cancers, and cancer in general. The majority of apps targeted breast cancer (46.8%, 138/295) (Figures 3 and Figure 4).

### Application Purpose and Content

The reported app purpose was predominantly to raise awareness about cancer (32.2%, 95/295) or provide information about cancer (26.4%, 78/295). A considerable proportion of apps were designed to support fundraising efforts (12.9%, 38/295) or promote a charitable organization raising awareness or funds for cancer (10.2%, 30/295). A minority of apps aimed to assist in the prevention (2.0%, 6/295), early detection (11.5%, 34/295), or management of cancer (3.7%, 11/295), and only 3 apps (1.0%, 3/295) enabled users to communicate with or learn from other cancer survivors. Apps affiliated with a commercial company were more likely to have an informational purpose, while apps affiliated with a not-for-profit were more likely to have a fundraising, awareness, or promotional purpose (χ^2^_2_=13.3, *P*=.001). Application purpose and features are summarized in Table 1.

The majority of apps utilized the visual media capability of the smartphone to deliver content. The top three multimedia formats were: visual media-only (36.7%, 108/295), text-only (28.9%, 83/295), and a combination of text and visual media (22.6%, 65/295). The apps that contained only visual media consisted of themed backgrounds or icons (eg, pink ribbons), which were intended to raise awareness of cancer. Many apps (31.5%, 93/295) used a combination of multi-media content. (Data not shown.)

Screenshots of representative apps from each category are shown in Multimedia Appendix 2.

**Table 1:** Primary function and features of cancer apps (n=295).

Purpose	Description	Features^a^	Apps, n (%)
Awareness	Images, information, or games to raise awareness about cancer or issues related to cancer	1. Cancer-themed wallpaper, icons, fonts, or programs (eg, pink ribbon background)	95 (32.2)
		2. Basic information to raise awareness about cancer	
		3. Cancer-themed games (eg, chemo drug shooting at cancer cells)	
		4. Interactive activities to raise awareness about cancer (eg, trivia about cancer)	
Disease and treatment information	Educational information, in some cases supplemented by visual or video media, regarding: disease, diagnosis, symptoms, treatment, prevention, screening, alternative therapy, behavior management, cancer terminology, psychosocial issues, or up-to-date news.	1. eBook	78 (26.4)
		2. Newsfeed	
		3. Glossary of terms	
		4. Directory of information with search functionality	
		5. Instructional images or videos	
Fundraising	Tools to raise funds for cancer or support management of fundraising efforts.	1. Promote fundraising efforts (eg, Twitter, Facebook)	38 (12.9)
		2. Monitor fundraising progress	
		3. Update personal fundraising page	
		4. Fundraising event information	
		5. Donate funds	
Early detection	Educational information, skills training, and tools to assist in the detection of cancer.	1. Information, images, or videos on how to screen	34 (11.5)
		2. Monitor screening results (eg, notes or pictures)	
		3. Medical screening reminder	
		4. Forms or image capture tools with built-in GPS locator of screening or cancer centers	
		5. Image capture tool with algorithm that calculates skin cancer risk	
Promote an organization	Encourage awareness about a charitable organization raising awareness and funds for cancer or providing support to people affected by cancer.	1. Newsfeeds	30 (10.2)
		2. Information about the organization	
		3. Organizational contact information	
		4. Direct links to contact the organization (eg, hyperlink to a contact form on a website)	
Disease management	Information and tools to manage the medical, behavioral, or emotional aspects of cancer	1. Appointment tools: reminder/ organizer, medical team contact list, tools to prepare questions, tools to store lab results, journal for notes, tools to track medical expenses	11 (3.7)
		2. Self-monitoring/ tracking tools: physical symptom tracker, psychosocial symptom tracker, medication tracker	
		3. Communication (eg, email)	
Prevention	Educational information, skills training, and tools to prevent cancer.	1. Grocery list of foods that may prevent cancer	6 (2.0)
		2. Recipes based on recommend cancer-fighting foods	
		3. Quiz on reducing behavior and dietary risk factors	
		4. Information and images on how to exercise	
Peer support	Tools to communicate with or learn from the experiences of other cancer patients.	1. Asynchronous communication tools	3 (1.0)
		2. GPS locator of social network members	
		3. Text or audio-based cancer survivor stories	

^a^The features listed here are illustrative of the main app content in each category.

### Prevention, Early Detection, and Disease Management Apps

Six apps (2.0%, 6/295) reported that their purpose was to assist in the prevention of cancer by promoting healthy lifestyle behaviors. Five of these apps aimed to promote healthy eating behaviors; one app promoted exercise. These apps targeted breast cancer (n=4), prostate cancer (n=1), and cancer in general (n=2). The healthy eating apps offered educational information and skill-building tools to assist in the performance of healthy eating behaviors. Features included lists of cancer preventative foods, recipes based on recommended foods, and tips on healthy eating practices. The exercise app offered instruction on how to perform exercises to promote increased circulation in breast tissue.

Thirty-four apps (11.5%, 34/295) reported that their purpose was to assist in the early detection of cancer. The majority of these apps targeted breast cancer (n=17) or skin cancer (n=9), followed by hematologic cancers (eg, leukemia, lymphoma, myeloma; n=3), testicular cancer (n=2), cervical (n=1), and colorectal (n=1). Most of the former apps offered practical guidance on how to perform self-exams, reminders for self-exams or screening appointments, and features to track assessment results. A handful of apps offered cancer risk scores based on completion of a questionnaire. Skin cancer detection apps included tools that captured and tracked images of skin lesions and generated cancer risk scores based on built-in algorithms.

Eleven apps (3.7%, 11/295) provided tools to support the management of cancer. The majority of these apps were not specific to a particular cancer type (n=7), three were tailored for breast cancer (2 of which also targeted colorectal cancer), and one was tailored for prostate cancer. These apps offered a combination of tools to assist in the management of (1) medical appointments (eg, appointment organizers or reminders, team contact lists, question list builders, note taking, recoding of lab results, tracking of medical costs), or (2) self-monitoring of symptoms or medication consumption (eg, using forms or journaling features). One app was focused entirely on assisting in the preparation of question lists to guide conversations with health professionals, including an inventory of over 100 typical questions, and tools to add a question, build question lists, and record answers.

### Evaluation

No app reported an evaluation of any form in their store description.

The search strategy yielded 594 articles. After independent review by two authors, none of these were deemed eligible. We did not find an evaluation of a cancer-focused smartphone app. But we did find evaluations of three unique mobile device interventions for cancer: a symptom management system to facilitate monitoring of toxicity symptoms in patients undergoing chemotherapy, which produces tailored self-management feedback and alerts the care team alerts when alarming symptoms arise [17-19]; a generic symptom assessment system that prompts patients to perform and record assessments and forwards completed assessments to clinicians for review [20]; and a problem-solving skills system for parents of children with cancer with prompts to perform and log problem-solving activities [21]. One of these was designed for a basic mobile phone and the others were PDA-based interventions.

## Discussion

### Principal Findings

In summary, a total of 295 unique apps were identified across the four leading smartphone markets. The majority of apps targeted breast cancer or cancer in general and aimed to raise awareness about cancer or provide educational information about cancer. Most apps affiliated with a non-profit organization were free. However, most apps did not report their organizational affiliation. Last, a systematic review of the health literature found no evaluations of cancer-focused smartphone applications.

This study demonstrates, as have others [6-9], the increasing number of native health apps that are available to the public. The majority of reviewed apps were available on Android or iPhone smartphones, which is unsurprising given the dominance of Google and Apple in the smartphone market [18]. Some early reviews of health apps focused exclusively on iPhone apps [6,8,9]. This study demonstrates the importance of considering Google’s Android platform, which accounted for 75% of the worldwide smartphone market share as of the third-quarter of 2012 [18].

Despite increasing interest in mobile phones as platforms for the delivery of health behavior-changing interventions, this study suggests that the cancer apps available in the app stores, on their own, have limited potential value in this regard. It is well recognized that information alone is insufficient to change behavior, particularly when complex behavior change is the aim [19]. To be effective, health promotion efforts must also: teach the self-management skills necessary to translate that knowledge into effective practices; build a sense of self-efficacy or confidence in performing the behaviors; and create the social supports necessary for the initiation and maintenance of the desired behavior [20]. Yet, the majority of identified apps focused exclusively on raising awareness or delivering information about cancer. Only 17.2% (51/295) of apps provided information in combination with skill-building tools to assist in the performance of preventive, detection, or self-management behaviors. Similarly, Rosser and Eccleston [6] found that the majority of pain-related apps were designed to deliver information about pain and its treatment, with little integration of features to promote coping or self-management behaviors. In contrast, Chomutare et al [7] found an under-representation of education in their review of diabetes apps, which were rich in self-management features. Their review, on the other hand, was restricted to apps that had a blood glucose self-monitoring component, which could have excluded educational apps.

Overall, the reviewed cancer apps did not take advantage of the smartphone’s technical capabilities. For example, the main feature offered by the subset of apps that aimed to support preventative or self-management behaviors was health information tracking, often referred to as self-monitoring. This was primarily achieved through native journaling applications custom-designed to support logging of appointment information or health-related behaviors. Self-monitoring has been shown to be an effective health behavior intervention, particularly for weight loss [21]. However, the effort involved in tracking one’s activities can be a significant barrier to adoption and sustained use. Mobile phones can reduce the effort involved in self-monitoring by using photos to document complex behaviors and using sensors connected wirelessly to measuring devices that automatically log behaviors and physiologic states [2]. Most of the apps included in this review relied on textual entry or touch screen completion of predetermined response options. A handful of the skin cancer detection apps enabled users to document and track their skin lesions using the phone’s built-in camera and three of the question-building apps enabled users to audio record health professionals’ responses. There were no apps that used automated sensing for tracking.

Although there are fewer clear physiologic indicators for cancer that are amenable to user self-collection, mobile sensing platforms could assist in the automated logging of symptoms (eg, fatigue, pain, nausea) or health behaviors such as exercise. The threat of regulation, which is costly and time-consuming, could have discouraged app developers from using the smartphone’s technical capabilities, particularly automated sensing. Regulatory bodies in the United States and the European Union are increasing scrutiny over mobile apps, with the United States opting for a larger scope [22]. On September 25, 2013, the US Food and Drug Administration (FDA) issued guidelines for the oversight of mobile medical apps that meet the regulatory definition of “device” and that (1) are intended to be used as an accessory to an FDA-regulated medical device (eg, an app that could enable a health professional to view a medical image), or (2) transform a mobile platform into a regulated medical device (eg, apps that use sensors to measure and track vital signs) [23]. However, some mobile sensing apps that meet the definition of a medical device will not require FDA review, for example, apps that allow users to collect (electronically or manually entered) blood pressure data and share these data through email or upload it to a personal or medical health record. Although these FDA guidelines were not available at the time the search was conducted, draft guidelines of a similar nature were available and could have influenced app developers.

Effective self-management requires effective communication with and support of the health care team [19,20]. Only a few apps included features that could facilitate communication with the health care team. These were limited tools to identify and prioritize questions to ask your doctor and journaling apps to take notes during medical appointments. In their review of pain apps, Rosser and Eccleston [6] found no apps that facilitated communication with the health care team, which they attributed to the lack of involvement of health professionals in app development. Health professionals can also support self-management efforts through regular assessment of their patients’ health status and providing encouragement to perform healthy behaviors, as well as problem-solving support [19]. Involvement of health professionals has also been shown to increase adherence to Web-based interventions [24]. Mobile phones can keep health professionals informed of the patient’s condition and progress and facilitate health professional-patient interactions, through remote coaching, remote symptom monitoring, and automated feedback [2]. As discussed, the mobile smartphone’s connectivity to the Internet facilitates remote monitoring efforts by enabling user’s data to be uploaded to a server as soon as they are captured, allowing for early detection of critical events. For example, Kearney et al [25] developed a PDA-based symptom management system to facilitate monitoring of toxicity symptoms in patients undergoing chemotherapy. The system uses phone-based questionnaires to assess six chemotherapy-related symptoms (nausea, vomiting, fatigue, mucositis, hand-foot syndrome, and diarrhea). Patients’ responses are uploaded to a server and the system generates specific strategies for managing the particular symptoms. If the software determines that the symptoms are alarming, the system generates an alert that is sent to the health care team. Reported benefits of the system based on patients’ evaluations include: improved communication with health professionals, improvements in management of symptoms, and feeling reassured their symptoms were being monitored. While apps that enable patients to self-record their symptoms and to upload that information to a server for health professionals are considered medical devices according to the FDA, the agency has decided to waive regulation of these apps.

The reviewed apps also failed to fully take advantage of the smartphone’s social networking capabilities. Only three apps enabled users to connect with similar others to exchange information and support. Two of these apps enabled users to post questions and responses to other users of the app in the form of a mobile community of support, and the third consisted of a book of survivors’ experiences with the disease. None of the prevention and disease management apps included features that enabled the exchange of supportive information with other users of the app. Similarly, Chomutare et al [7] documented a lack of social media functionality in their review of diabetes apps. Most diabetes apps that claimed to include social media features only provided a link to the device’s group page in social networking sites such as Twitter and Facebook. Online health communities have repeatedly demonstrated their value in bringing together cancer patients and survivors to exchange information and support [26]. While the exact mechanisms by which social relationships affect health remains unclear, nearly 30 years of research has consistently demonstrated that they have a powerful effect on physical and mental health and may extend survival [27]. Social support is also a critical factor in the initiation and maintenance of health behaviors [20]. Mobile phones have the potential to connect users with their support networks anytime and anywhere.

The lack of evidence of app effectiveness and description of the procedures or data sources (eg, evidence, theory, or user-centered design) used to develop the app is also concerning. All five previous reviews of health apps have raised this concern [6-10], two of which demonstrated discrepancies between information generated on smartphone applications and evidence-based guidelines [7,8]. Pandey et al [9] found that significantly more iPhone apps designed for health care professionals had scientifically valid information compared to those designed for patients (96% vs 32%). Although evaluating the credibility of app content was out of scope, and in our view sufficiently addressed in previous reviews, the “iEAT” app in Multimedia Appendix 2 provides a good example of questionable content that lacks cited source material. Our study found that the majority of apps failed to report their organizational affiliation (64.1%, 189/295).

Last, compared to cancer incidence in Canada [28], there is a nearly two-fold over-representation of the percent distribution of breast cancer apps (eg, 45% of apps compared to 26.1% new cases of breast cancer), and a considerable under-representation of prostate, lung, and colorectal apps. In part, this is likely because a large proportion of the apps were intended as awareness-raising tools as opposed to addressing perceived or real need and there is greater charitable activity around breast cancer. The breast cancer fundraising movement is one of the largest and most successful social movements, which other groups seek to emulate [29]. In addition, the overwhelming majority of investment in cancer research in Canada (27%) is focused on breast cancer [30].

There is a need for a whitelist of regulatory body-approved (when necessary), scientifically evaluated, and consumer-recommended mobile health apps. Our study found several apps for melanoma detection that would likely fall under FDA regulation. These apps aim to aid users in determining their melanoma risk by analyzing a digital image of the user’s lesion based on built-in algorithms. A use case study of the accuracy of four of these types of apps to correctly classify 60 melanoma and 128 benign control lesions, found the results to be highly variable: 3 of the 4 apps incorrectly classified 30% or more of the melanoma lesions as unconcerning [10]. These types of apps have the potential to cause distress and harm if they provide the patient with advice that is misleading. The FDA maintains a list of apps on its website that have been approved by the agency [23]. The National Health Service (NHS) in the United Kingdom has gone one step further in producing a Library of Health Apps [31] that are reviewed by the NHS to ensure that they are clinically safe and rated by consumers. There are also CONSORT-eHealth reporting guidelines for randomized controlled trials of Web-based and mobile health interventions [32]. Currently lacking is a synthesis of this information for consumers, reporting standards for app store descriptions, and a set of criteria to aid consumers in selecting health apps. This information could be beneficial to developers, funders, and health professionals as well, and may improve the development of future apps and stimulate work in neglected areas.

### Limitations

Our study had certain limitations. First, the review of the smartphone apps was restricted to the commercial descriptions of the apps in the online stores; as a result, certain apps may have been overlooked. Second, a considerable proportion of apps included in this review did not report their release dates, content source, or organizational affiliation in the store description. It is possible that some of this missing information is documented within the app. Third, the search results are dependent on the terms included in the search strategy and the search engines used. We attempted to overcome this limitation by choosing common terms, including multiple smartphone markets, as well as conducting the health literature review. Fourth, we conducted the searches in the Canadian and French mobile markets and, as a result, may have missed cancer apps available in the markets of other countries. Last, our selection criteria (eg, restricting to apps with “cancer” in the title or store description) likely excluded many prevention-related apps. Moreover, we intentionally excluded apps that were not focused on cancer or intended for people affected by cancer. Thus, the apps in the prevention category are likely not representative of all cancer prevention apps.

### Conclusions

Overall, this study found a considerable number of cancer-focused apps, available to consumers, of unknown utility and effectiveness. Although mobile devices offer remarkably low-cost, real-time ways to encourage preventive strategies, monitor a host of behaviors, symptoms and physiological indicators of disease, and provide interventions, the evidence base in support of these apps is lacking. Future efforts should focus on improving and consolidating the evidence on the utility, safety, and effectiveness of mobile cancer apps into a whitelist for public consumption.

## Multimedia Appendix 1

Mobile app review - literature search strategy.

## Multimedia Appendix 2

Examples of representative iPhone apps from each category.

## Abbreviations

API: application programming interfaces
FDA: Food and Drug Administration
PDA: personal digital assistant
