# Supplementary material for: A Lot of Action, But Not in the Right Direction: Systematic Review and Content Analysis of Smartphone Applications for the Prevention, Detection, and Management of Cancer
Source: J Med Internet Res. 2013 Dec 23;15(12):e287. doi: 10.2196/jmir.2661 (PMC3875901; doi:10.2196/jmir.2661)
Supplement: Supplementary file 1 [file jmir_v15i12e287_app1.pdf]

Multimedia Appendix 1: Literature Search Strategy [MEDLINE version]

1. [HEDGE - Cellular or Mobile or Handheld or Wireless Technologies]
2. Cellular Phone/
3. exp microcomputers/ or computers, handheld/
4. blackberry.mp.
5. blackberrys.mp.
6. bluetooth.mp.
7. (car adj (phone or phones)).mp.
8. (cell* adj phone?).mp.
9. (cell* adj telephon*).mp.
10. electr* medical assistant*.mp.
11. (handheld adj1 comput*).mp.
12. (hand-held adj1 comput*).mp.
13. (handheld adj1 devic*).mp.
14. (hand-held adj1 devic*).mp.
15. iphone?.mp.
16. ipod?.mp.
17. (mobile adj1 devic*).mp.
18. (mobile adj1 phon*).mp.
19. (mobile adj1 techn*).mp.
20. (mobile adj telephon*).mp.
21. multimedia messag*.mp.
22. multi-media messag*.mp.
23. "palm os".mp.
24. palmpilot?.mp.
25. palm pilot?.mp.
26. "palm pre".mp.
27. (palmtop adj1 comput*).mp.
28. (palm-top adj1 comput*).mp.
29. (pda adj1 comput*).mp.
30. personal digital assist*.mp.
31. short messag*.mp.
32. smartphon*.mp.
33. smart phon*.mp.
34. (sms adj2 alert*).mp.
35. (sms adj2 remind*).mp.
36. (sms adj2 text*).mp.
37. (text adj2 alert*).mp.
38. (text adj1 messag*).mp.
39. (text adj2 remind*).mp.
40. txt msg*.mp.
41. voice mail*.mp.
42. voicemail*.mp.
43. (voice adj1 messag*).mp.
44. walkie-talkie?.mp.
45. wifi?.mp.

46. wi-fi?.mp.
47. wireless*.mp.
48. skype.mp.
49. voip?.mp.
50. (voice adj1 internet*).mp.
51. ipad.mp.
52. ipads.mp.
53. android.mp.
54. windows mobil*.mp.
55. imedical app*.mp.
56. (medical adj2 app).mp.
57. (medical adj2 apps).mp.
58. (health* adj2 app).mp.
59. (health* adj2 apps).mp.
60. (mobile adj3 app).mp.
61. (mobile adj3 apps).mp.
62. (mhealth adj4 app).mp.
63. (mhealth adj4 apps).mp.
64. or/1-63
65. Neoplasms/
66. 64 and 65
67. limit 66 to english language
