# Supplementary material for: A Lot of Action, But Not in the Right Direction: Systematic Review and Content Analysis of Smartphone Applications for the Prevention, Detection, and Management of Cancer
Source: J Med Internet Res. 2013 Dec 23;15(12):e287. doi: 10.2196/jmir.2661 (PMC3875901; doi:10.2196/jmir.2661)
Supplement: Supplementary file 2 [file jmir_v15i12e287_app2.pdf]

Multimedia Appendix 2: Examples of representative iPhone apps from each category

Category and App Name	Screen Shots
Awareness<br>*Pink Ribbon Breast Cancer Wallpaper*	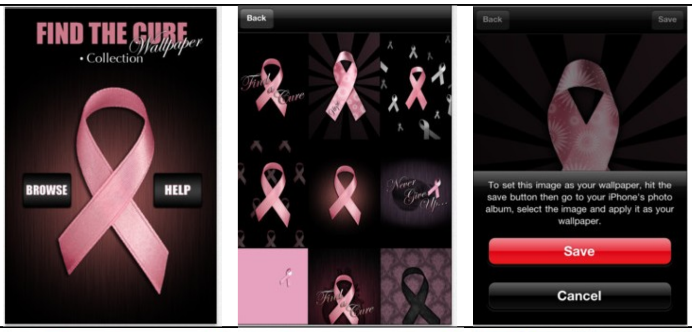
Information<br>*Cancer iOncolex*	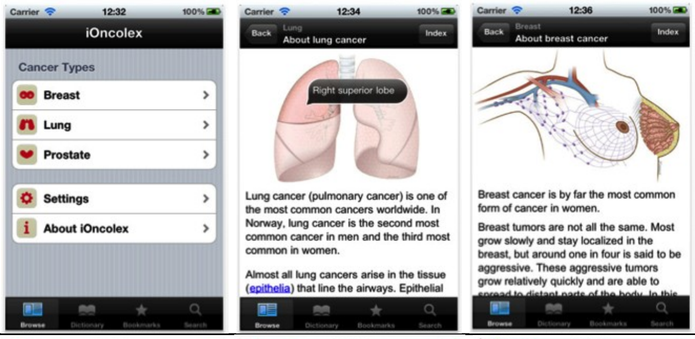
Fundraising<br>*The Ride to Conquer Cancer*	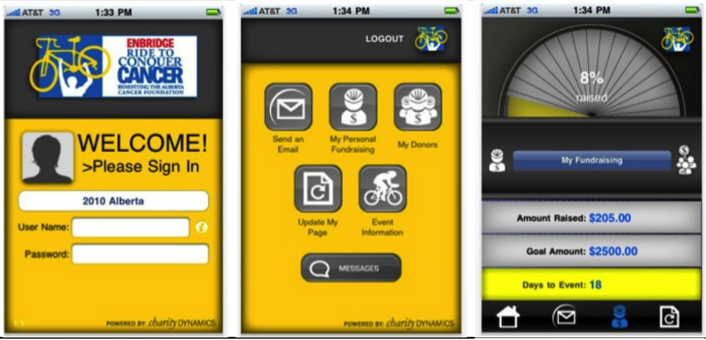
Promote an Organization<br>*Conquer Cancer Foundation*	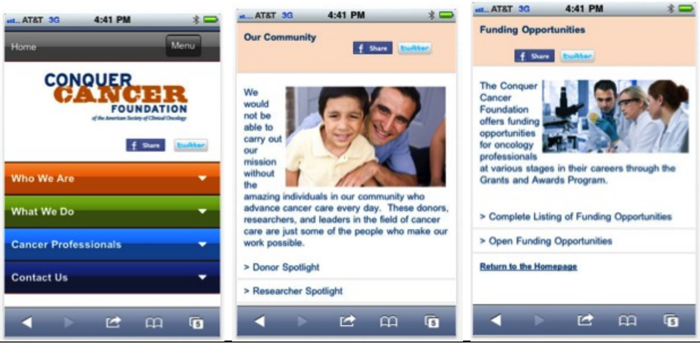

Early Detection<br>*Skin Cancer- The Most Accurate Skin Cancer Detector on iPhone*	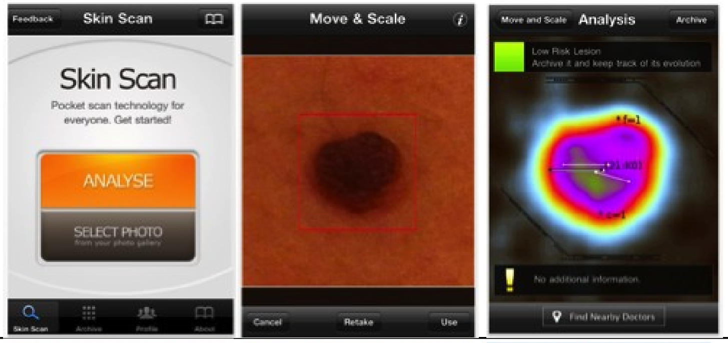
Disease Management<br>*Cancer Net*	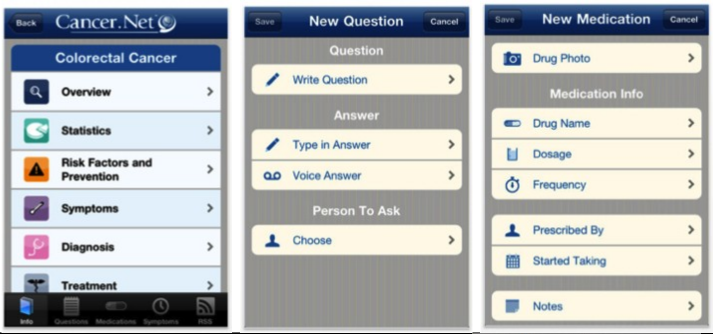
Prevention<br>*iEat for Life: Prostate Cancer*	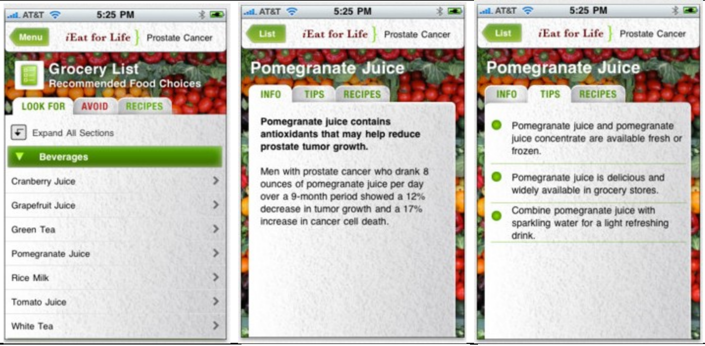
Peer Support<br>*Breast Cancer Connect*	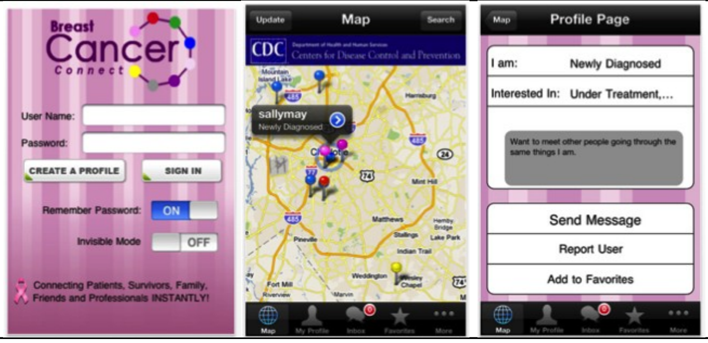
